# Genetic Diversity in Introduced Golden Mussel Populations Corresponds to Vector Activity

**DOI:** 10.1371/journal.pone.0059328

**Published:** 2013-03-22

**Authors:** Sara Ghabooli, Aibin Zhan, Paula Sardiña, Esteban Paolucci, Francisco Sylvester, Pablo V. Perepelizin, Elizabeta Briski, Melania E. Cristescu, Hugh J. MacIsaac

**Affiliations:** 1 Great Lakes Institute for Environmental Research, University of Windsor, Windsor, Ontario, Canada; 2 Research Center for Eco-Environmental Sciences, Chinese Academy of Sciences, Beijing, China; 3 Museo Argentino de Ciencias Naturales “Bernardino Rivadavia”, Buenos Aires, Argentina; 4 Australian Centre for Biodiversity, School of Biological Sciences, Monash University, Melbourne, Australia; 5 Universidad de Buenos Aires, Departamento de Ecología, Genética y Evolución, Buenos Aires, Argentina; 6 Consejo Nacional de Investigaciones Científicas y Técnicas, Buenos Aires, Argentina; 7 Fisheries and Oceans Canada, Great Lakes Laboratory for Fisheries and Aquatic Sciences, Burlington, Ontario, Canada; University of Canterbury, New Zealand

## Abstract

We explored possible links between vector activity and genetic diversity in introduced populations of *Limnoperna fortunei* by characterizing the genetic structure in native and introduced ranges in Asia and South America. We surveyed 24 populations: ten in Asia and 14 in South America using the mitochondrial cytochrome *c* oxidase subunit I (COI) gene, as well as eight polymorphic microsatellite markers. We performed population genetics and phylogenetic analyses to investigate population genetic structure across native and introduced regions. Introduced populations in Asia exhibit higher genetic diversity (*H*_E_ = 0.667–0.746) than those in South America (*H*_E_ = 0.519–0.575), suggesting higher introduction effort for the former populations. We observed pronounced geographical structuring in introduced regions, as indicated by both mitochondrial and nuclear markers based on multiple genetic analyses including pairwise Ф_ST_, *F*_ST_, Bayesian clustering method, and three-dimensional factorial correspondence analyses. Pairwise *F*_ST_ values within both Asia (*F*_ST_ = 0.017–0.126, *P* = 0.000–0.009) and South America (*F*_ST_ = 0.004–0.107, *P* = 0.000–0.721) were lower than those between continents (*F*_ST_ = 0.180–0.319, *P* = 0.000). Fine-scale genetic structuring was also apparent among introduced populations in both Asia and South America, suggesting either multiple introductions of distinct propagules or strong post-introduction selection and demographic stochasticity. Higher genetic diversity in Asia as compared to South America is likely due to more frequent propagule transfers associated with higher shipping activities between source and donor regions within Asia. This study suggests that the intensity of human-mediated introduction vectors influences patterns of genetic diversity in non-indigenous species.

## Introduction

Blackburn et al. [1] proposed a unified framework for biological invasions that incorporates both distinctive stages for species moving between native habitats and those they are introduced into, and barriers between stages that serve to reduce overall invasion success. Differences among non-indigenous species (NIS), the vectors that spread them, and environmental characteristics of donor and recipient regions magnify the complexity of studying biological invasions [2]. Studies of population genetic structure of NIS have proven invaluable to our understanding of the invasion process and, in particular, to evolutionary aspects of invasions [3]–[5]. However, rapid and complex dynamics of human-mediated invasions can limit the applicability of genetic methods, which are mostly predicated on the existence of an equilibrium between key factors driving evolution (e.g. mutation, drift, selection). Therefore, it is essential to appreciate such limitations when studying genetics of introduced species, and to ask questions that can be answered using available resources [6].

The distribution and genetic structure of introduced populations can exhibit complex patterns [2]. A modern view of biological invasions recognizes that eroded genetic diversity [7]–[8] is not ubiquitous among introduced populations, as numerous studies have documented similar or increased genetic diversity owing to multiple introductions and/or high propagule pressure [9]–[12]. Propagule pressure refers to the number of individuals introduced to a region, and consists primarily of the number of introduction events (i.e. propagule number) and the number of individuals introduced per event (i.e. propagule size) [13]–[14]. Both components can affect genetic diversity of introduced populations. High propagule size may enhance establishment probability by lessening demographic stochasticity and the severity of genetic bottlenecks [14]–[15]. Increased propagule number diminishes the degree of environmental stochasticity and can increase the occurrence of admixture from different source populations [16]–[18]. Admixed populations may thus present with similar or even higher genetic diversity than any single native population [12], [15], [17], [19]–[20].

Genetic variation in introduced populations also depends on the structure of the source population [21]–[22]. For instance, introduced populations of the zebra mussel *Dreissena polymorpha* in North America exhibit lower but not severely diminished haplotype diversity relative to putative source populations in the Black Sea [23]. Some introduced populations appear to possess genotypes better adapted to changing environments than native ones, and they may become highly invasive in the invaded habitat [12]. Novel genotypes may appear in introduced populations owing to hybridization of different lineages seeded from divergent source populations or to hybridization with native species [21], [24].

The nature of transmitting vector can define the extent of propagule pressure and the genetic composition of introduced populations received from the source region [25]. Ships’ ballast water and hull fouling are recognized as major vectors in aquatic human-mediated invasions [26]–[30]. Areas receiving significant numbers of ship visits are at higher risk of biological invasions due to both ballast water discharge and/or hull fouling [31]–[32]. Certain life history characteristics may enhance the ability of species to invade [33]. For example, planktonic species (i.e. holoplankton) or those with planktonic life stage (i.e. meroplankton) have a higher chance of interfacing with a transport vector and of being carried to new region relative to species with strictly sessile, benthic life histories. Also, meroplanktonic species benefit from different types of transmitting vectors during their life cycle.

In this study, we explore genetic consequences of global spread of the golden mussel *Limnoperna fortunei*, a freshwater mytilid native to mainland China, Korea, Cambodia, Indonesia, Laos, and Vietnam [34]–[38]. The mussel was reported in Hong Kong in 1965, followed by Japan and Taiwan in late 1980s [38]–[39], and then in South America in 1991 in Argentina’s Río de la Plata estuary [40]. *Limnoperna* thereafter expanded its distribution very rapidly into Uruguay, Paraguay, Brazil and Bolivia, traveling an average of 240 km per year [41].

Japan, Taiwan, Korea, and parts of China are considered at high risk for biological invasion on the basis of ship traffic volume, while the opposite is true for South America [32]. In addition to possible introduction of *L. fortunei via* discharged ballast water [42], evidence suggests a second possible introduction pathway. It is possible that *L. fortunei* was introduced to Japan *via* aquaculture as a ‘fellow traveler’ with stocked Asian clams *Corbicula fluminea* imported from China [43]. This means *L. fortunei* could be introduced in Asia *via* at least two possible vectors (i.e. ballast water and aquaculture), whereas only a single vector (i.e. ballast water) appears possible for invasions in South America. Consequently the likelihood of admixed introduced populations should, be lower in South America than in Asia. This pattern serves as the basis of our first hypothesis: introduced populations of *L. fortunei* in South America will be genetically impoverished relative to introduced ones in Asia. Introduced populations in Asia (Japan and Taiwan) are separated by geographical barriers (marine water) whereas introduced South American populations have spread upstream from the putative initial invasion site in the Río de la Plata estuary along the Paraná, Uruguay, and Paraguay rivers. This pattern serves as the basis of our second hypothesis: gene flow is more limited among introduced Asian populations than among South American ones, and, as a result, genetic differentiation is more pronounced among introduced Asian populations. To test these hypotheses we used the cytochrome *c* oxidase subunit I (COI) gene and microsatellite markers to study genetic structure of this species in both continents.

## Materials and Methods

### Ethics Statement

No specific permits were required for the described field studies in Asia and South America. The species collected is an invasive pest in South America, Japan, and Taiwan and is not protected throughout this range. Sampling points did not include protected or private lands.

### Sample Collection, DNA Extraction and PCR

**Figure 1:**
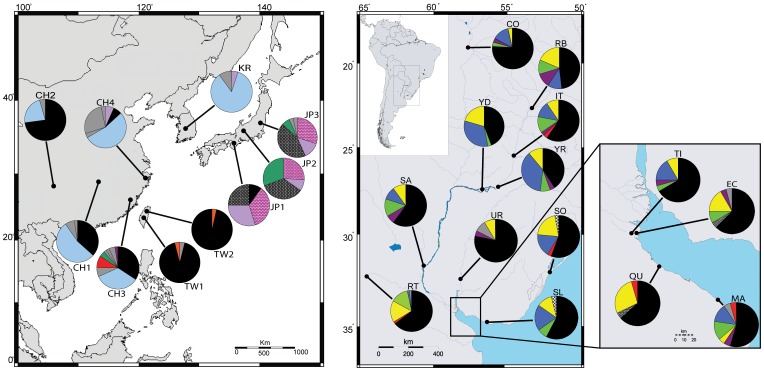
Haplotype distribution and frequency map for *Limnoperna fortunei*. Sampling sites and distribution of mitochondrial cytochrome *c* oxidase subunit I (COI) haplotypes for the native and introduced *L. fortunei* populations in Asia and South America. Site IDs as per Table 1. Different colors refer to different haplotypes. Private haplotypes that are not shared have similar color.

*Limnoperna fortunei* was sampled from 24 locations in Asia and South America, distributed across both native and introduced regions (Figure 1). Samples were collected from ten locations in Asia, including four from mainland China (native range), three from Japan, one from Korea (native range), and two from Taiwan, as well as 14 locations in South America covering the invaded range in the Paraná-Uruguay delta and the Río de la Plata estuary.

Genomic DNA was extracted from a piece of posterior abductor muscle using the protocol of Elphinstone et al. [44]. A fragment of the COI gene was amplified using species-specific forward primer, Limno-COIF1 [45], and universal reverse primer, HCO2198 [46]. PCR was performed with an initial denaturing at 94°C for 4 minutes followed by 5 cycles of 94°C for 50 s, 60°C for 50 s, 72°C for 60 s, 35 cycles of 94°C for 50 s, 55°C for 50 s, 72°C for 60 s, and a final elongation at 72°C for 5 minutes. Purified PCR products were sequenced using the reverse primer and BigDye Terminator 3.1 chemistry with an ABI 3130XL automated sequencer (Applied Biosystems, Foster City, CA). All sequences that contained ambiguous sites were subsequently sequenced with the forward primer. We genotyped all populations at eight microsatellite loci [47]. Fragment analysis was performed using an ABI 3130XL automated sequencer with GeneScan™–500 LIZ™ size standard. Allele sizes were decided using GeneMapper version 3.7 (Applied Biosystems). In order to validate the scoring results, we re-ran random samples from multiple plates.

### MtDNA Analysis

Sequences were aligned using CodonCode Aligner 2.0 (CodonCode Corporation, Dedham, MA) and then manually edited. The possibility of doubly uniparental inheritance (DUI) of mitochondrial genome observed in other Mytilidae species was tested and ultimately excluded according to the method described by Gillis et al. [4]. The best fit evolutionary model was estimated using MRMODELTEST version 3.7 [48] with the Bayesian Information Criterion (BIC). Bayesian phylogenetic analysis was conducted with MRBAYES version 3.2 [49]. Trees were sampled every 500 generations for five million generations and the first 25% of the sampled trees were discarded as burn-in. A sequence for the green mussel *Perna perna* was used as the outgroup (Genbank accession no: EF493941).

A parsimony haplotype network with 95% connection probability [50] was generated using TCS v.1.21 [51] to resolve relationships among haplotypes. Sixteen COI sequences of *L. fortunei* from Japan were retrieved from GenBank (Accession nos. AB520611– AB520627) and included in phylogenetic analyses.

The number of haplotypes (*n*), haplotype diversity (*h*), and nucleotide diversity (*π*), were estimated using DnaSP 5.0 [52]. Genetic differentiation between populations was determined by Ф_ST_ with the Tamura-Nei substitution model implemented in ARLEQUIN version 3.1 [53]. Sequential Bonferroni corrections were used to adjust the significance level for multiple comparisons [54]. Hierarchical genetic structure was assessed using analysis of molecular variance (AMOVA) based on 10,000 random permutations in ARLEQUIN. Populations were grouped based on *a priori* expectations according to their geographical origin into two groups: i) all South American populations versus all populations from Asia; and ii) based on their geographic region within Asia (samples were divided as native: China, Korea and introduced: Taiwan, Japan) and within South America (samples were divided based on river basins into six regions: I Upper Paraguay (CO), II Paraná River (RB, IT, YR, YD and SA), III Uruguay River (UR), IV Río Tercero (RT), V Sao Gonçalo (SO), and VI Paraná Delta - Río de la Plata (EC, TI, QU, SL and MA).

### Microsatellite Analysis

Genetic diversity indices including the number of alleles (*A*), allelic frequency (*F*), allelic richness (*A*_r_), observed heterozygosity (*H*_O_), and expected heterozygosity (*H*_E_) were measured using FSTAT v.2.9.3 [55]. Allelic richness (*A*_r_), which is an estimate of allelic diversity adjusted by the lowest sample size, was calculated using Fstat. Genetic differentiation among populations was examined by *F*_ST_ using ARLEQUIN. Similar to Ф_ST,_ sequential Bonferroni corrections were used to adjust the significance level for multiple comparisons [54]. Due to recent criticisms on the use of *F*_ST_ and the interpretation of population differences, we also calculated one of the corrected *F*_ST_ –like indices Jost’s D [56] using the online software SMOGD [57]. We also performed AMOVA on microsatellite data using the same criteria used for COI dataset to group populations.

To further investigate population genetic structure of *L. fortunei,* we employed a Bayesian clustering method using STRUCTURE v. 2.3.3 [58]. The range of possible clusters (*K*) was tested from one to 24 (total number of populations) and ten independent runs for each *K* value were set at 10^6^ Markov chain Monte Carlo (MCMC) iterations, with an initial burn-in of 10^5^. We followed the method of Evanno et al. [59] to determine the value of *K*. We assessed possible hierarchical genetic structure by conducting separate Bayesian analyses on populations from South America (*K* from 1 to 14), and Asia (*K* from 1 to 10), plus Taiwan and Japan (*K* from 1 to 5). A three-dimensional factorial correspondence analysis (3D-FCA) was performed using GENETIX v. 4.05 [60]. Contrary to STRUCTURE, this method does not assume Hardy- Weinberg Equilibrium and was used to validate results obtained from STRUCTURE.

## Results

Analysis of the whole 510-bp alignment obtained from 697 individuals resulted in 32 mtDNA haplotypes for COI (GenBank accession Nos. HQ843794-HQ843806, HQ84373808-09, JX177086-JX177102). All haplotypes were recovered after we used only female mussels (F-type haplotypes), suggesting that doubly uniparental inheritance (DUI) is not characteristic of *L. fortunei*. Of the 16 Japanese haplotypes retrieved from GenBank, eight were identical to those detected in this study. The haplotype frequency map revealed a high level of geographic structure (Figure 1). Native populations in mainland China and Korea and introduced ones in Asia (Taiwan and Japan) and South America, had similar haplotypes in each region with only a few haplotypes shared among these groups. One haplotype (Lfm03) was common in all but three Asian populations (KR, JP2, and JP3) (Figure 1, Table 1), although haplotype frequency differed in each region. We identified 12 haplotypes in South America, with three (Lfm01, Lfm04–05) common to most of the populations (Figure 1). Twenty-three haplotypes were found in Asia, three of which were shared with South America (Lfm02–03, and Lfm06; Table 1, Figure 1).

**Table 1:** Sampling details and genetic diversity indices for mitochondrial and microsatellite markers for *Limnoperna fortunei*.

ID	Collection site and Country	Latitude	mtDNA					Microsatellite				
		Longitude	*N*	*n*	Haplotype Code	*h*	*Π*	*N*	*A*	*A*_r_	*H*_O_	*H*_E_
**Asia**												
TW1	Sun Moon Lake, Taiwan	23.842°	26	3	Lfm03, Lfm08,	0.151	0.0013	29	11.4	7.3	0.4670	0.6673
		120.872°			Lfm19							
TW2	Shiandau, Fusing	24.806°	28	2	Lfm03, Lfm08	0.071	0.0006	43	12.6	7.3	0.4286	0.7432
	Township, Taiwan	121.252°										
JP1	Daido intake station, Yodo	34.745°	20	4	Lfm09, Lfm03,	0.753	0.0079	14	6.9	6.2	0.4524	0.7460
	River, Japan	135.551°			Lfm20––21							
JP2	Yahagi River, Toyota,	35.112°	23	4	Lfm09, Lfm20–21,	0.637	0.0056	48	10.1	6.1	0.4265	0.7150
	Japan	137.194°			Lfm27							
JP3	Lake Ohshio, Tomioka,	36.223°	30	6	Lfm09, Lfm20–21,	0.743	0.0079	30	11.1	7.1	0.4129	0.7210
	Japan	138.876°			Lfm27–29							
KR	Korea Institute of Water	36.401°	20	3	Lfm11, Lfm21,	0.279	0.0006	30	14.5	9.5	0.3985	0.8576
	and Environment, Korea	127.413°			Lfm26							
CH1	Lake Poyang, China	29.185°	41	4	Lfm03, Lfm11,	0.587	0.0068	45	18.0	8.8	0.4994	0.8059
		116.014°			Lfm24–25							
CH2	Pengxi River, Yunyang	30.948°	22	3	Lfm03, Lfm11,	0.437	0.0050	22	10.0	7.3	0.5280	0.7263
	County, China	108.680°			Lfm30							
CH3	Xiongjiang, Minqing	26.327°	44	9	Lfm02–03, Lfm 06,	0.766	0.0084	44	13.1	7.1	0.5175	0.7006
	County, China	118.744°			Lfm11–12, Lfm27,							
					Lfm31–33							
CH4	Luohe River, Zhejiang	28.878°	30	6	Lfm03, Lfm11,	0.655	0.0083	30	11.0	7.1	0.4529	0.7015
	Province, China	121.165°			Lfm21, Lfm35–37							
**South America**												
CO	Corumbá, Brazil	−18.997°	29	5	Lfm01–05	0.416	0.0021	30	6.8	6.0	0.2214	0.5366
		−57.654°										
RB	Río Baía, Alto Rio Paraná,	−22.686°	27	5	Lfm01–05	0.724	0.0059	33	6.9	5.9	0.2285	0.5474
	Brazil	−53.253°										
IT	Itaipú Hydroelectric Power	−25.408°	32	6	Lfm01–06	0.625	0.0033	30	7.9	6.9	0.2802	0.6067
	Reservoir, Brazil	−54.590°										
YR	Yabebiry River, Misiones,	−27.297°	27	5	Lfm01–05	0.704	0.0037	28	6.9	6.3	0.1392	0.5575
	Argentina	−55.543°										
YD	Yaciretá Dam, Brazil,	−27.471°	34	4	Lfm01, Lfm03–05	0.677	0.0029	29	6.3	5.8	0.1283	0.5578
	Paraguay and Argentina	−56.704°										
SA	Setubal Lagoon, Santa Fe,	−31.635°	30	5	Lfm01–05	0.618	0.0042	34	7.4	6.3	0.2413	0.5764
	Argentina	−60.681°										
SO	Sao Gonçalo Channel,	−31.811°	34	5	Lfm03–06, Lfm10	0.631	0.0034	34	6.8	5.9	0.2153	0.5628
	Brazil	−52.388°										
UR	Uruguay River, Colón,	−32.152°	23	4	Lfm02–03, Lfm05,	0.387	0.0025	26	5.3	5.0	0.2046	0.5843
	Argentina	−58.188°			Lfm17							
RT	Río Tercero Dam,	−32.213°	59	6	Lfm01, Lfm03–06,	0.546	0.0022	30	7.6	6.5	0.1795	0.5648
	Córdoba, Argentina	−64.473°			Lfm13							
EC	Del Este Channel, Buenos	−34.346°	24	6	Lfm01–03, Lfm05,	0.594	0.0041	40	7.8	6.6	0.1784	0.5835
	Aires, Argentina	−58.519°			Lfm07, Lfm14							
TI	Luján River, Tigre, Buenos	−34.415°	24	5	Lfm01–05	0.540	0.0068	40	9.0	7.1	0.1893	0.6312
	Aires, Argentina	−58.578°										
QU	Quilmes, Buenos Aires,	−34.716°	22	4	Lfm03, Lfm05–07	0.541	0.0028	40	7.6	6.7	0.1807	0.6090
	Argentina	−58.214°										
SL	Santa Lucía River,	−34.810°	26	5	Lfm01, Lfm03–05,	0.634	0.0038	30	7.1	6.4	0.2417	0.5945
	Canelones, Uruguay	−56.431°			Lfm10							
MA	Magdalena, Buenos Aires,	−35.013°	22	7	Lfm01–06, Lfm16	0.688	0.0036	34	6.5	5.5	0.2163	0.5390
	Argentina	−57.536°										
Total			697	32		0.604	0.0033	793	311	6.1	0.2670	0.6025

*N*, sample size for different molecular markers in different populations; *n*, number of haplotypes; *h*, haplotype diversity; *π*, nucleotide diversity; *A*, number of alleles; *A*_r_: allelic richness; *H*_O_ and *H*_E_, mean observed heterozygosity and expected heterozygosity computed at eight microsatellite loci.

**Figure 2:**
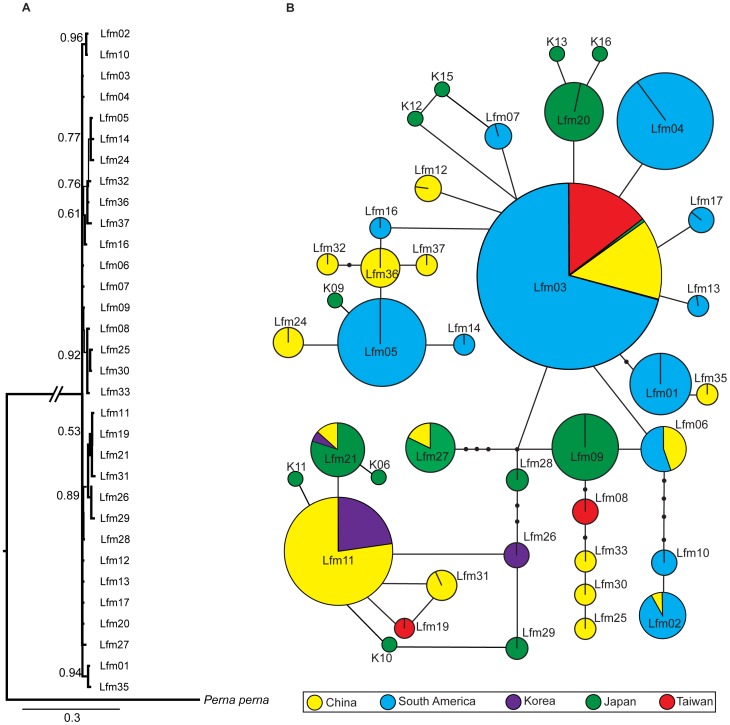
Phylogenetic analyses of *Limnoperna fortunei*. Bayesian inference tree (A) based on the mitochondrial cytochrome *c* oxidase subunit I (COI) haplotypes. Numbers are posterior probabilities recovered by Bayesian analysis, and only values above 50% are shown. COI haplotype parsimony network (B) for *L. fortunei* in Asia and South America. Haplotype names as per Table 1. Haplotypes are indicated by circles, the size of which corresponds to frequency. Missing or unsampled haplotypes are indicated by black circles. Colors indicate different geographical regions from which the sample was collected. Haplotype names starting with *K* correspond to extra sequences from Japan retrieved from Genbank.

The Bayesian phylogenetic tree showed a shallow structure lacking apparent phylogeographic structure (Figure 2A). We observed a similar pattern in the statistical parsimony haplotype network, with a star-shaped topology and only a few mutation steps among haplotypes (Figure 2B).

We detected the highest number of haplotypes (*n* = 9) in population CH3 sampled from mainland China, while only two haplotypes were recovered from population TW2 collected from Taiwan. Mean haplotype diversity (*h*) and nucleotide diversity (*π*) in Chinese populations were 0.611 and 0.007, respectively, higher than those observed in invaded areas. Comparable values for South America were 0.595 and 0.004, while those in introduced populations in Asia were 0.439 and 0.004, respectively. Populations from Taiwan (TW1 and TW2) exhibited the lowest haplotype and nucleotide diversity (0.111 and 0.001, respectively; Table 1). Pairwise values of Ф_ST_ ranged from 0.005–0.945 in Asia, 0.000–0.077 in South America, and 0.003–0.867 between these two continents. These values were non-significant in South America, while most were significant in Asia or between the continents (Table 2). There was more genetic variance within (71.3%) than among (18.3%) populations (AMOVA; *P*<0.001 and 0.020, respectively; Table 3).

**Table 2:** Estimates of population genetic differentiation based on the mitochondrial cytochrome oxidase subunit I (mtDNA COI) gene (pairwise Ф_ST_, above diagonal) and microsatellite markers (pairwise *F*_ST_, below diagonal) for *Limnoperna fortunei*, across the introduced range in South America.

	TW1	TW2	JP1	JP2	JP3	KR	CH1	CH2	CH3	CH4	CO	RB	IT	YR	YD	SA	SO	UR	RT	EC	TI	QU	SL	MA
TW1	****	−0.022	0.254	0.284	0.219	0.906	0.399	0.102	0.212	0.427	0.011	0.064	0.028	0.097	0.131	0.031	0.075	0.003	0.052	0.059	0.018	0.134	0.037	0.032
TW2	0.025	****	0.315	0.327	0.264	0.945	0.450	0.170	0.256	0.476	0.012	0.081	0.036	0.117	0.157	0.041	0.095	0.012	0.059	0.080	0.028	0.179	0.051	0.042
JP1	0.055	0.059	****	0.112	0.008	0.544	0.116	0.057	0.037	0.143	0.280	0.185	0.248	0.251	0.274	0.224	0.242	0.240	0.325	0.207	0.238	0.231	0.235	0.230
JP2	0.096	0.077	0.046	****	0.034	0.659	0.299	0.178	0.168	0.313	0.292	0.187	0.257	0.259	0.277	0.232	0.243	0.248	0.332	0.209	0.248	0.228	0.243	0.241
JP3	0.088	0.089	0.083	0.069	****	0.610	0.217	0.095	0.098	0.247	0.243	0.158	0.212	0.222	0.236	0.195	0.199	0.205	0.274	0.173	0.206	0.186	0.203	0.201
KR	0.086	0.070	0.071	0.093	0.126	****	0.284	0.680	0.376	0.262	0.878	0.742	0.825	0.819	0.832	0.796	0.818	0.867	0.856	0.805	0.849	0.856	0.831	0.835
CH1	0.043	0.039	0.037	0.070	0.095	0.047	****	0.147	0.021	0.005	0.426	0.341	0.401	0.396	0.411	0.379	0.391	0.393	0.464	0.360	0.392	0.378	0.387	0.388
CH2	0.050	0.055	0.059	0.096	0.122	0.071	0.039	****	0.026	0.187	0.145	0.100	0.127	0.149	0.173	0.111	0.136	0.116	0.177	0.109	0.119	0.148	0.121	0.120
CH3	0.042	0.046	0.061	0.085	0.103	0.086	0.040	0.029	****	0.060	0.245	0.187	0.232	0.236	0.254	0.214	0.229	0.215	0.290	0.201	0.218	0.219	0.220	0.217
CH4	0.042	0.051	0.066	0.095	0.105	0.096	0.048	0.042	0.017	****	0.445	0.337	0.412	0.402	0.413	0.387	0.395	0.404	0.485	0.356	0.401	0.369	0.394	0.394
CO	0.285	0.250	0.266	0.261	0.268	0.216	0.223	0.263	0.253	0.269	****	0.024	−0.013	0.014	0.061	−0.009	0.033	−0.007	0.010	0.037	−0.033	0.111	−0.017	−0.020
RB	0.302	0.262	0.291	0.276	0.285	0.233	0.229	0.289	0.279	0.286	0.055	****	−0.005	0.014	0.032	−0.021	0.001	0.007	0.037	−0.019	−0.006	0.020	−0.009	−0.011
IT	0.251	0.221	0.234	0.234	0.241	0.180	0.189	0.230	0.229	0.235	0.037	0.056	****	0.003	0.033	−0.029	0.012	−0.000	0.005	−0.003	−0.027	0.054	−0.028	−0.035
YR	0.276	0.234	0.252	0.233	0.252	0.197	0.205	0.238	0.225	0.245	0.037	0.107	0.042	****	−0.008	0.007	0.010	0.054	0.014	0.043	−0.013	0.090	−0.020	−0.001
YD	0.296	0.249	0.281	0.263	0.275	0.201	0.215	0.274	0.254	0.271	0.032	0.071	0.057	0.085	****	0.041	−0.011	0.074	0.007	0.033	0.017	0.042	−0.000	0.046
SA	0.319	0.283	0.300	0.293	0.294	0.234	0.242	0.299	0.283	0.299	0.031	0.036	0.031	0.048	0.045	****	0.014	−0.006	0.019	−0.010	−0.024	0.048	−0.024	−0.035
SO	0.305	0.259	0.283	0.256	0.273	0.218	0.221	0.278	0.265	0.282	0.059	0.029	0.049	0.083	0.087	0.039	****	0.018	−0.007	−0.008	−0.004	−0.006	−0.012	0.023
UR	0.305	0.265	0.279	0.268	0.285	0.218	0.229	0.270	0.264	0.283	0.045	0.045	0.047	0.064	0.063	0.038	0.029	****	0.018	−0.002	−0.015	0.046	−0.001	0.000
RT	0.304	0.267	0.283	0.258	0.286	0.234	0.233	0.278	0.272	0.284	0.032	0.038	0.050	0.069	0.025	0.018	0.029	0.016	****	0.018	−0.013	0.040	−0.015	0.019
EC	0.273	0.224	0.253	0.234	0.242	0.187	0.191	0.240	0.225	0.244	0.028	0.071	0.027	0.024	0.054	0.030	0.082	0.072	0.056	****	0.003	−0.022	−0.004	0.006
TI	0.305	0.259	0.284	0.261	0.280	0.222	0.221	0.276	0.258	0.276	0.044	0.065	0.039	0.030	0.056	0.019	0.046	0.040	0.037	0.035	****	0.056	−0.036	−0.030
QU	0.281	0.242	0.257	0.249	0.255	0.199	0.209	0.256	0.243	0.259	0.004	0.050	0.024	0.032	0.033	0.022	0.059	0.048	0.029	0.015	0.020	****	0.040	0.077
SL	0.306	0.267	0.291	0.278	0.281	0.225	0.213	0.286	0.273	0.287	0.073	0.024	0.046	0.090	0.080	0.029	0.008	0.052	0.040	0.085	0.043	0.063	****	−0.028
MA	0.296	0.257	0.272	0.248	0.272	0.215	0.223	0.262	0.256	0.271	0.029	0.073	0.029	0.047	0.076	0.049	0.037	0.034	0.025	0.061	0.045	0.037	0.066	****

Underlined numbers indicate statistical significance after sequential Bonferroni adjustments. Population identifications as per Table 1. Horizontal and vertical double lines separate populations from Asia and South America.

**Table 3:** Analysis of molecular variance (AMOVA) for *L. fortunei.*

Source of variation	Sum ofsquares	Variancecomponents	Percentageof variation	*P*-value
**mtDNA**				
Group 1 (Asia and South America)				
Among groups	130.64	0.365	21.8	<0.001
Among groups	130.64	0.365	21.8	<0.001
Among populations within groups	162.63	0.219	13.0	<0.001
Within populations	736.00	1.094	65.2	<0.001
Group 2 (region based)				
Among groups	206.46	0.282	18.3	0.002
Among populations within groups	86.81	0.158	10.3	<0.001
Within populations	735.90	1.093	71.3	<0.001
**Microsatellite**				
Group 1 (Asia and South America)				
Among groups	477.14	0.602	21.5	<0.001
Among populations within groups	226.22	0.125	4.5	<0.001
Within populations	3239.0	2.074	74.0	<0.001
Group 2 (region based)				
Among groups	598.68	0.432	13.9	<0.001
Among populations within groups	198.94	0.166	5.3	<0.001
Within populations	3551.11	2.520	80.8	<0.001

Populations are grouped based on their geographical distribution; group 1 (10 populations from Asia, and 14 populations from South America) and group 2 (native regions in Asia: China, Korea, introduced regions in Asia: Japan, Taiwan and regions in South America: (CO), (RB, IT, YR, YD, SA), (UR), (RT), (SO), (EC, TI, QU, SL, MA)). *P*-values for all groups indicate significant differences.

We successfully genotyped 793 individuals from 24 populations at eight microsatellite loci, resulting in a total of 311 alleles. Mean allelic richness (*A*_r_) ranged from 5 to 9.5, while mean expected heterozygosity (*H*_E_) and observed heterozygosity (*H*_O_) ranged from 0.518 to 0.858, and from 0.128 to 0.528, respectively (Table 1, Table S1). Mean expected heterozygosity (*H*_E_) and mean allelic richness (*A*_r_) were higher in Asia as compared to South America (for *H*_E_: U = 0, Z = 4.07, *P*<0.0001; for *A*_r_: U = 17.5, Z = 3.04, *P* = 0.0012). Many loci (150 of 192) deviated from Hardy-Weinberg equilibrium (HWE) and all exhibited heterozygosity deficiency (Table S1). Genetic differentiation based on pairwise *F*_ST_ ranged from 0.015 to 0.319 among all population comparisons. *F*_ST_ values within Asia (0.067) and South America (0.046) were lower than those between the continents (0.257). Almost all *F*_ST_ values were significant in Asia and between Asia and South America while most of the *F*_ST_ values were non-significant in South America. In Asia, pairwise *F*_ST_ values were lower inside each geographic region (0.036 in mainland China, 0.025 in Taiwan, and 0.066 in Japan) as compared to the overall value (0.067) for the continent (Table 2). In South America, the overall pairwise *F*_ST_ was 0.046, which is significantly lower than that of Asia (U = 200.5, Z = 2.89, *P* = 0.0019). We observed relatively high genetic differentiation between neighbouring populations in South America, for example, *F*_ST_ = 0.072 between EC and UR (separated by ∼ 50 km), while some geographically distant populations exhibited relatively low *F*_ST_ values, for example, 0.004 between CO and QU (separated by ∼ 2000 km). Similar to *F*_ST_ values, Jost’s D values were lower among populations in South America (D = 0.00–0.074) than in Asia (D = 0.024–0.404), and between Asia and South America (D = 0.351–0.726) (Table 4). Genetic variance was greater within (80.8%) than among (13.9%) populations (AMOVA, *P*<0.001; Table 3).

**Table 4:** Estimates of population genetic differentiation (corrected *F*_ST_ –like index, Jost’D) based on microsatellite markers for *Limnoperna fortunei*, across the introduced range in South America.

	TW1	TW2	JP1	JP2	JP3	KR	CH1	CH2	CH3	CH4	CO	RB	IT	YR	YD	SA	SO	UR	RT	EC	TI	QU	SL	MA
TW1	****																							
TW2	0.039	****																						
JP1	0.088	0.127	****																					
JP2	0.200	0.171	0.071	****																				
JP3	0.163	0.200	0.123	0.131	****																			
KR	0.274	0.304	0.245	0.391	0.404	****																		
CH1	0.124	0.102	0.104	0.225	0.265	0.259	****																	
CH2	0.132	0.114	0.122	0.235	0.292	0.320	0.091	****																
CH3	0.079	0.108	0.119	0.213	0.199	0.328	0.086	0.030	****															
CH4	0.086	0.130	0.144	0.205	0.213	0.319	0.123	0.056	0.024	****														
CO	0.525	0.463	0.523	0.411	0.496	0.536	0.522	0.488	0.462	0.482	****													
RB	0.624	0.559	0.633	0.537	0.602	0.612	0.555	0.616	0.598	0.589	0.021	****												
IT	0.476	0.418	0.511	0.409	0.472	0.508	0.463	0.451	0.452	0.435	0.033	0.047	****											
YR	0.544	0.484	0.522	0.389	0.504	0.519	0.521	0.453	0.448	0.471	0.023	0.072	0.024	****										
YD	0.597	0.558	0.599	0.494	0.593	0.610	0.581	0.568	0.564	0.552	0.041	0.031	0.052	0.055	****									
SA	0.553	0.500	0.543	0.420	0.511	0.543	0.526	0.518	0.514	0.503	0.017	0.026	0.028	0.039	0.037	****								
SO	0.631	0.575	0.612	0.454	0.589	0.626	0.538	0.570	0.587	0.616	0.044	0.014	0.044	0.058	0.049	0.039	****							
UR	0.531	0.463	0.511	0.411	0.504	0.500	0.478	0.421	0.444	0.464	0.034	0.032	0.040	0.048	0.066	0.025	0.024	****						
RT	0.559	0.514	0.546	0.414	0.521	0.559	0.528	0.508	0.500	0.502	0.007	0.011	0.039	0.044	0.031	0.010	0.018	0.015	****					
EC	0.552	0.484	0.536	0.439	0.507	0.518	0.532	0.465	0.467	0.476	0.017	0.042	0.039	0.013	0.041	0.012	0.072	0.031	0.021	****				
TI	0.555	0.482	0.530	0.397	0.508	0.530	0.494	0.469	0.473	0.471	0.031	0.044	0.029	0.014	0.036	0.015	0.033	0.020	0.031	0.020	****			
QU	0.552	0.484	0.525	0.398	0.500	0.525	0.524	0.471	0.467	0.470	0.000	0.025	0.028	0.019	0.033	0.012	0.052	0.037	0.015	0.011	0.018	****		
SL	0.726	0.662	0.687	0.502	0.659	0.693	0.594	0.636	0.647	0.652	0.040	0.019	0.058	0.068	0.051	0.020	0.003	0.031	0.018	0.066	0.026	0.037	****	
MA	0.528	0.472	0.534	0.351	0.508	0.548	0.498	0.403	0.418	0.458	0.019	0.046	0.026	0.041	0.074	0.039	0.011	0.026	0.022	0.046	0.032	0.023	0.026	****

Population identifications as per Table 1. Horizontal and vertical double lines separate populations from Asia and South America.

**Figure 3:**
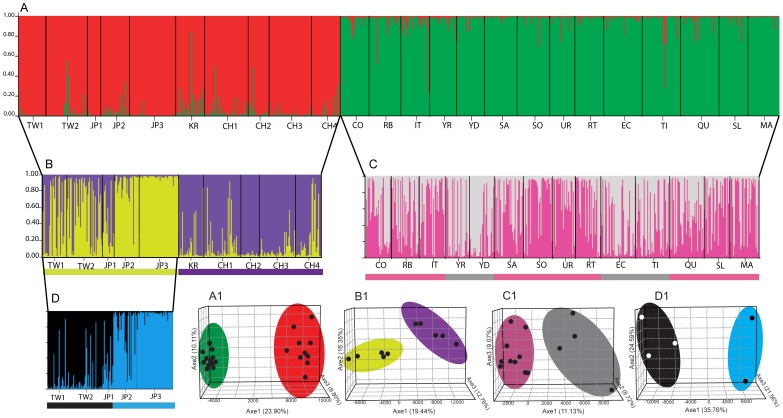
Bayesian inference population genetic structure of *Limnoperna fortunei*. Bayesian clustering of *L. fortunei* based on eight polymorphic microsatellites in all 24 populations (A), populations collected from the native range in Asia (B), introduced populations in South America (C), and introduced populations in Asia (D). Each genotype is represented by a thin vertical line, with proportional membership in different clusters indicated by different colors. Bold vertical lines separate collection sites, with site identifications indicated below the plot. Site identification as per Table 1. Three-dimensional factorial correspondence analysis (A1– D1) corresponding to the Bayesian clustering of *L. fortueni*.

Bayesian clustering analysis revealed two clusters when all populations were considered, corresponding to Asian and South American groupings (Figure 3A). Likewise, only two groupings (*K* = 2) were supported when Asia (Figure 3B) and South America (Figure 3C) were analyzed separately. Within Asia, populations from mainland China and Korea were grouped separately from those collected from Taiwan and Japan (Figure 3A). Populations from Taiwan and Japan could, in turn, be subdivided into two clusters (*K* = 2). Two populations from Taiwan (TW1, TW2), and one Japanese population (JP1) were grouped together, while the other two Japanese populations (JP2 and JP3) were clustered into another group (Figure 3D). South American clusters showed a genetically discontinuous distribution: some geographically distant populations were grouped in the same cluster, whereas some proximal ones were assigned to different clusters (Figure 3C). The 3D-FCA revealed consistent results with the pattern obtained from Bayesian clustering method (Figure 3A1–D1).

## Discussion

We used both mtDNA (COI) and nuclear markers (microsatellites) to contrast the geographical distribution of genetic diversity of *Limnoperna fortunei* in Asia and South America. Three major findings emerge from this survey. First, introduced populations in South America exhibited lower genetic diversity relative to comparable ones in Asia. Second, genetic variation was geographically structured in introduced populations on both continents. Third, our results suggest that more than one introduction event might have occurred in each of Asia and South America. Higher genetic diversity in the former is consistent with higher propagule pressure associated with introduction vectors from neighboring source regions.

Expected heterozygosity (0.667–0.746) for introduced populations in Japan and Taiwan was higher than that in introduced South American ones (0.519–0.575). Both groups exhibited lower expected heterozygosity as compared to native populations in mainland China and Korea (0.701–0.858). The high number of haplotypes at the COI locus recovered from Japan, coupled with relatively high heterozygosity, suggests that *L. fortunei* has been introduced more than once and/or in large inocula (i.e. high propagule pressure). This conclusion is consistent with Tominaga *et al*.’s (2009) [61] findings. It is possible that *L. fortunei* was introduced to Japan *via* aquaculture as a ‘fellow traveler’ with stocked Asian clams, *C. fluminea*, imported from China [43]. According to the Japan Fish Traders Association (JFTA, 2010) and the World Health Organization (WHO publications 2010), Japan is the largest importer of clams in Asia. China and Korea are the main sources of clams imported to Japan, and both countries host native populations of *L. fortunei* [35], [37]. For example, during the period 1989–2011, more than 76% of freshwater clams imported to Japan originated from China or Korea [62]. Trade between Asian countries continues to grow, and with it the risk of further spread of *L. fortunei* to neighboring countries [63].

We observed a low number of mtDNA haplotypes in Taiwan (*n* = 3), but rather high level of allelic richness (*A*_r_ = 7.3) and heterozygosity (*H*_E_ = 0.7052) at microsatellite loci (Table 1). MtDNA has smaller effective population size (*N*_e_) relative to nuclear DNA owing to its maternal inheritance and haploid nature. Therefore, the mitochondrial genome is expected to be more sensitive to bottleneck events than the nuclear genome [64]. Similar genetic patterns have been observed in other non-indigenous species, such as the ascidian *Ciona intestinalis* [2]. Two possible processes - sweepstakes reproductive success and dramatic demographic changes during translocation - could lead to stronger signatures of genetic drift on mtDNA [2]. Taiwan, a major bivalve market in Asia for oysters and scallops, imports animals mainly from the USA, Canada and Japan (WHO publications, 2010). These imported species are mainly marine [65], thus the risk of *L. fortunei* introduction from Japan through aquaculture appears to be low. *L. fortunei* has not yet been reported in USA or Canada. While aquaculture remains a possible vector for introduction to Taiwan, it is more likely active in Japan. The low haplotype diversity observed in Taiwan could be also result from inhospitable environmental conditions in primary introduction areas. It is also possible that the putative source population carries similar level of genetic diversity. Korea was also represented by a low number of haplotypes, and high nuclear allelic diversity, relative to native populations in mainland China (Table 1). However, since only one Korean population was surveyed, we suspect that this pattern might be the product of low sample size.

Heterozygosity deficit was found in 77.6% of all analyzed microsatellite loci, including native populations in mainland China and Korea (Table S1). This pattern has been reported in other invasive, freshwater bivalves, including quagga mussels (*D. rostriformis bugensis)* and zebra mussels (*D. polymorpha*) [65]–[69]. Several factors including Wahlund effect, inbreeding, selection, and null alleles could contribute to a heterozygosity deficiency. We observed high PCR amplification success rate for all loci examined, suggesting that null alleles were likely not a major factor responsible for the heterozygosity deficit. Given that planktonic free-swimming larvae of *L. fortunei* can be transported both up- and downstream through recreational boating, natural inland currents, and seasonal flooding, temporal and/or spatial Wahlund effect could account for the heterozygosity deficit, although inbreeding and selection cannot be completely dismissed.

### Genetic Variation among Populations

In Asia, both mtDNA and microsatellite markers exhibited lower genetic differentiation within geographic regions as compared to that among regions. This finding is supported by a higher percentage of variance allocated to among groups as compared to among populations within groups (AMOVA; Table 3). Japanese populations showed relatively high genetic differentiation, indicative of some population structure. This might be the result of separation of introduced populations of *L. fortunei* in each geographic region by a saltwater dispersal barrier, with inter-region gene flow limited to human-mediated translocation of propagules. In addition, distinct sources of introduction can drive genetic differentiation among regions. We observed high genetic differentiation between South American and Asian populations (*F*_ST_ = 0.180 – 0.306). High genetic differentiation has been reported in other freshwater invasive mussels, including *D. polymorpha* (*F*_ST_ = 0.006 – 0.263) and *D. rostriformis bugensis* (*F*_ST_ = 0.008 – 0.267) [23]. Our Bayesian analyses revealed fine-scale genetic structuring in Japan. One population from Japan (JP1) exhibited more admixtures with the other cluster containing populations from mainland China and Korea as compared to the other Japanese populations (JP2 and JP3), suggesting two possible genetically distinct sources for the introduced populations surveyed in Japan. A previous COI haplotype survey failed to recover fine-scale population genetic structure in Japan [61]. Similar to JP1, populations from Taiwan (TW1, TW2) exhibited some admixture with the other cluster containing native populations. The low genetic differentiation between TW1 and TW2 indicates similar source(s) or high gene flow within Taiwan. Long-distance or “jump” dispersal of *L. fortunei*, to upstream areas due to ship-mediated translocation appears responsible for the patchy post-establishment spread of the species in South America [47], [70]. Higher genetic differentiation among introduced populations in Asia relative to those in South America may be linked to the presence of geographical barriers between countries in Asia (e.g. East Sea China and Sea of Japan) as well as to possible genetically distinct propagule sources. The heterozygosity deficiency found in the surveyed *L. fortunei* populations violated the HWE assumption for STRUCTURE analyses. However, similar results were observed using another method (i.e. 3D-FCA) without this assumption.

The parsimony network analysis and Bayesian phylogenetic reconstruction revealed a close relationship among haplotypes of *L. fortunei*. It appears that a recent geographic expansion of *L. fortunei* throughout its native distribution can explain of its low phylogeographic structure. This finding suggests that even in the native region, populations may still be expanding to areas not previously populated. Vector activity such as ship traffic between local ports in native region (e.g. mainland China and Korea) may have contributed to recent expansion of *L. fortunei* across its native range [71].

### Asia versus South America

**Figure 4:**
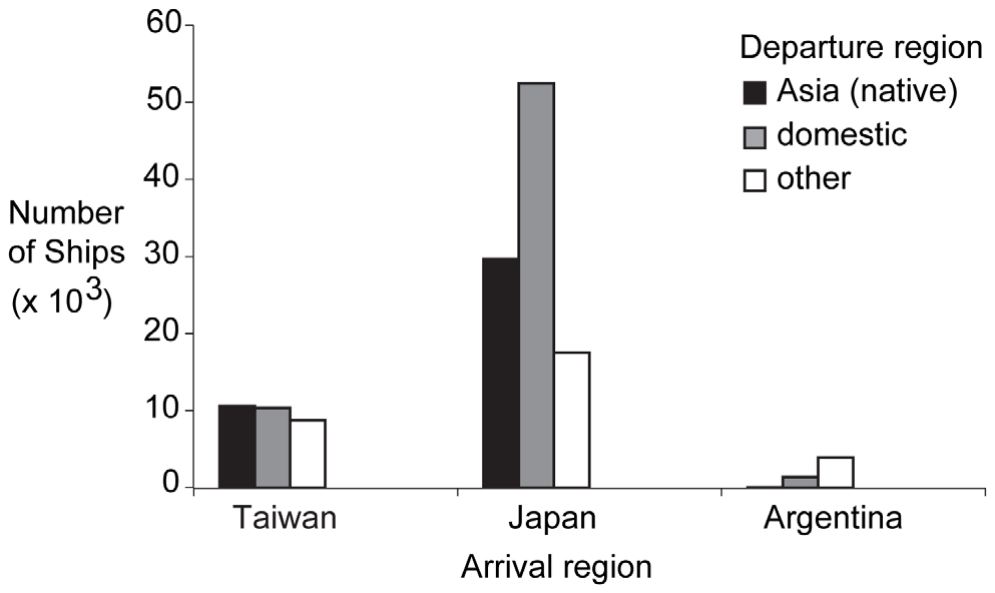
Ship traffic for Taiwan, Japan and Argentina. The total number of ships visiting each country is divided into: ships departing from countries considered native for *Limnoperna fortunei* (black bars), ships traveling between domestic ports in each country (grey bars), and ships departing from other global ports (white bars). Data is derived from supplementary information [71] provided by Lloyd’s Fairplay.

Our study suggests that introduced golden mussels carry less allelic diversity in South America than in Asia. A number of factors may contribute to this pattern. First, data from global ship traffic between 2005–2006 [71] documented higher ship traffic to ports in Japan and Taiwan relative to those in Argentina (Figure 4). For example, Japan received about 3 × 10^4^ ships from countries considered native for *L. fortunei*, whereas Argentina received only 26 ships. Second, Taiwan and especially Japan may benefit from an aquaculture transfers from adjacent Asian countries, notably mainland China. Third, given the comparatively short distance between Asian countries, hull fouling could effect local or regional spread. *L. fortunei* larger than 20 mm can tolerate anoxia for up to 18 days at 20°C [72], whereas it is unlikely that adults could survive ocean salinity (and hypoxia if valves are closed) while being transported on hull surfaces from Asia to eastern South America. The relatively high domestic traffic between local ports in each introduced country (Figure 4) suggests that shipping was a likely vector for secondary introduction of *L. fortunei* in these regions.

Although taxonomically distinct, *L. fortunei*, *D. polymorpha* and *D. rostriformis bugensis* share similar ecological and biological characteristics including planktonic larvae and a sessile, benthic adult stage. These life history traits suggest similar vectors could effect their human-mediated spread (i.e. ballast water and hull fouling). Also, golden mussel can be transported as a ‘fellow traveler’ in aquaculture [43], while this vector is possible but far less likely for either of the two dreissenid NIS [73]. Previous studies have assessed patterns of genetic diversity in populations of *D. polymorpha* and *D. rostriformis bugensis* in North America and Eurasia [23], [74]. Our survey of *L. fortunei* provides a good basis for comparison of the genetic characteristics of the three mussel species (Table S2). When using microsatellites, all three species have similar level of genetic diversity across native and introduced regions (Table S2). The higher number of COI haplotypes retrieved from *L. fortunei* (40 haplotypes) as compared to 11 found for *D. polymorpha* [75] and seven for *D. rostriformis bugensis* [76] suggests that the former species did not experience historical population fluctuations following colonization like the latter species. For *D. polymorpha*, the Great Lakes appear to have served as a ‘hub’ for subsequent expansion across North America [23]. Similarly, the Río de la Plata estuary appears to have served as a ‘staging hub’ for subsequent spread of introduced golden mussels through much of eastern South America.

## Conclusions

Our findings suggest that *L. fortunei*’s introduction in both Asia and South America likely involved multiple introductions and high propagule pressure, resulting in populations with high genetic diversity relative to sampled native populations in Asia. Introduced populations exhibiting lower genetic diversity (South America) likely received lower propagule pressure relative to those with higher diversity (Japan). Our genetic survey shows how human-mediated introduction of NIS can create genetic complexities across introduced locations. Our study evaluates possible links between vector activity and genetic composition of a nuisance NIS at a global scale, and highlights the utility of incorporating population genetics and vector activity data to understand species dispersal patterns [77]–[78].

## Supporting Information

Figure S1 **Values of Δ** ***K*** **calculated as in Evanno et al.** [**59**] **for detecting the biologically relevant clusters of** ***Limnoperna fortunei*** **collected from all 24 locations (A) and Asia (B).**
(TIF)

Table S1 Genetic diversity at eight microsatellite loci for the golden mussel, *Limnoperna fortunei*, sampled from 24 locations across the global range in East Asia and South America. *A*, number of alleles; *A*_r_, allele richness; *H*_O_, observed heterozygosity; *H*_E_, expected heterozygosity; *P*_HW_, exact *P*-value for Hardy-Weinberg equilibrium test. The significance after sequential Bonferroni correction was bolded.
(DOC)

Table S2 Comparison of microsatellite-based genetic features of the three highly invasive freshwater mussels, zebra mussel *Dreissena polymorpha*, quagga mussel *Dreissena rostriformis bugensis*, and golden mussel *Limnoperna fortunei.*
(DOC)
