# Supplementary figures and images for: Genetic Diversity in Introduced Golden Mussel Populations Corresponds to Vector Activity
Source: PLoS One. 2013 Mar 22;8(3):e59328. doi: 10.1371/journal.pone.0059328 (PMC3606440; doi:10.1371/journal.pone.0059328)

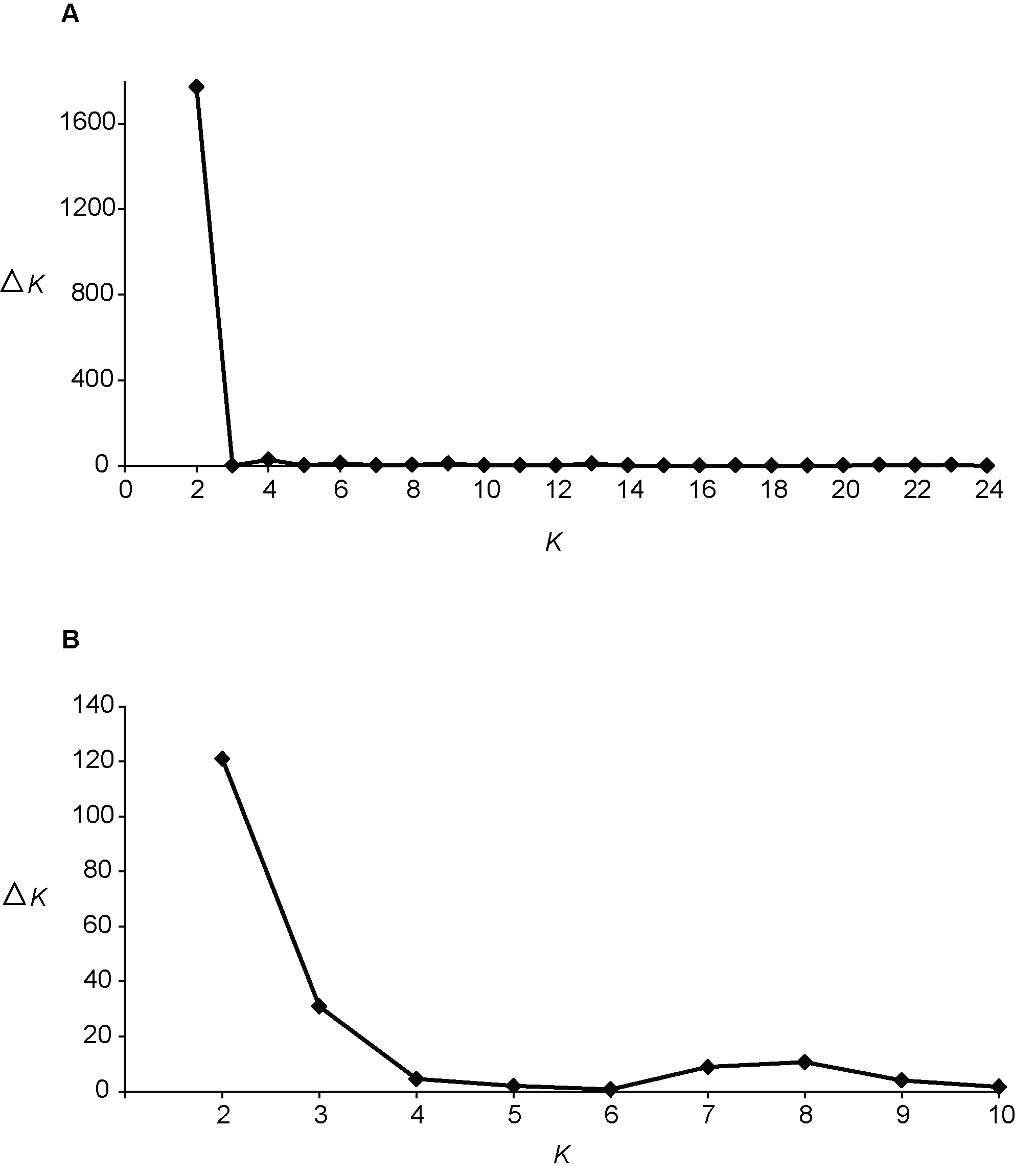

Supplement: Figure S1 — Values of Δ K calculated as in Evanno et al. [59] for detecting the biologically relevant clusters of Limnoperna fortunei collected from all 24 locations (A) and Asia (B). (TIF) [file pone.0059328.s001.tif]
