# Supplementary material for: Genetic Diversity in Introduced Golden Mussel Populations Corresponds to Vector Activity
Source: PLoS One. 2013 Mar 22;8(3):e59328. doi: 10.1371/journal.pone.0059328 (PMC3606440; doi:10.1371/journal.pone.0059328)
Supplement: Table S1 — Genetic diversity at eight microsatellite loci for the golden mussel, Limnoperna fortunei, sampled from 24 locations across the global range in East Asia and South America. A, number of alleles; A r, allele richness; H O, observed heterozygosity; H E, expected heterozygosity; P HW, exact P-value for Hardy-Weinberg equilibrium test. The significance after sequential Bonferroni correction was bolded. (DOC) [file pone.0059328.s002.doc]

**Table S1** Genetic diversity at eight microsatellite loci for the golden mussel, *Limnoperna fortunei*, sampled from 24 locations across the global range in East Asia and South America. A, number of alleles; *A*_r_, allele richness; *H*_O_, observed heterozygosity; *H*_E_, expected heterozygosity; *P*_HW_, exact *P*-value for Hardy-Weinberg equilibrium test. The significance after sequential Bonferroni correction

was bolded.

| ID | Index |  |  |  | Microsatellite loci | | |  |  | Average |
| --- | --- | --- | --- | --- | --- | --- | --- | --- | --- | --- |
|  |  | Lf04 | Lf06 | Lf07 | Lf19 | Lf21 | Lf22 | Lf23 | Lf38 |  |
| TW1 | *A*/*A*_r_ | 16/9.4 | 13/10.1 | 6/4.6 | 20/9.7 | 12/7.1 | 6/5.5 | 9/4.6 | 9/7.3 | 11.4/7.3 |
|  | *H*_O_ | 0.2963 | 0.5200 | 0.5833 | 0.5714 | 0.6897 | 0.4091 | 0.2500 | 0.4167 | 0.4670 |
|  | *H*_E_ | 0.8498 | 0.9127 | 0.6153 | 0.7468 | 0.6945 | 0.7950 | 0.3422 | 0.7775 | 0.6673 |
|  | *F*_IS_ | 0.66 | 0.44 | 0.05 | 0.24 | 0.01 | 0.49 | 0.27 | 0.47 | 0.33 |
|  | *P*_HW_ | **0.0000** | **0.0000** | 0.3822 | 0.0526 | **0.0122** | **0.0003** | 0.0086 | **0.0000** |  |
|  |  |  |  |  |  |  |  |  |  |  |
| TW2 | *A*/*A*_r_ | 21/11.9 | 11/83 | 9/6.1 | 13/6.3 | 28/11.3 | 8/6.1 | 5/3.3 | 6/5.5 | 12.6/7.3 |
|  | *H*_O_ | 0.4146 | 0.5128 | 0.5897 | 0.3721 | 0.4883 | 0.3947 | 0.2051 | 0.4516 | 0.4286 |
|  | *H*_E_ | 0.9301 | 0.8578 | 0.7369 | 0.5543 | 0.8627 | 0.7923 | 0.4829 | 0.7282 | 0.7432 |
|  | *F*_IS_ | 0.56 | 0.41 | 0.20 | 0.33 | 0.44 | 0.51 | 0.58 | 0.38 | 0.43 |
|  | *P*_HW_ | **0.0000** | **0.0000** | **0.0000** | **0.0000** | **0.0000** | **0.0000** | **0.0000** | **0.0018** |  |
|  |  |  |  |  |  |  |  |  |  |  |
| JP1 | *A*/*A*_r_ | 10/8.7 | 5/4.9 | 8/6.8 | 9/7.6 | 8/7.3 | 5/5.0 | 5/4.5 | 5/4.9 | 6.9/6.2 |
|  | *H*_O_ | 0.3077 | 0.4616 | 0.5385 | 0.4286 | 0.4286 | 0.3000 | 0.6923 | 0.4615 | 0.4524 |
|  | *H*_E_ | 0.8031 | 0.7354 | 0.6862 | 0.7064 | 0.8492 | 0.7316 | 0.7200 | 0.7354 | 0.7460 |
|  | *F*_IS_ | 0.63 | 0.38 | 0.22 | 0.40 | 0.51 | 0.60 | 0.04 | 0.38 | 0.40 |
|  | *P*_HW_ | 0.0000 | 0.0037 | **0.2363** | 0.0251 | 0.0025 | 0.0063 | 0.1917 | 0.0079 |  |
|  |  |  |  |  |  |  |  |  |  |  |
| JP2 | *A*/*A*_r_ | 17/10.6 | 9/6.1 | 18/11.0 | 14/7.0 | 13/7.6 | 10/6.8 | 3/2.9 | 5/4.4 | 11.1/7.1 |
|  | *H*_O_ | 0.6923 | 0.3333 | 0.3333 | 0.4615 | 0.2759 | 0.2593 | 0.6000 | 0.3479 | 0.4129 |
|  | *H*_E_ | 0.9005 | 0.7475 | 0.9001 | 0.5716 | 0.8221 | 0.7435 | 0.5808 | 0.5015 | 0.7210 |
|  | *F*_IS_ | 0.24 | 0.56 | 0.63 | 0.20 | 0.67 | 0.79 | -0.03 | 0.31 | 0.42 |
|  | *P*_HW_ | **0.0000** | **0.0000** | **0.0000** | 0.1513 | **0.0000** | **0.0000** | **0.0000** | **0.0128** |  |
|  |  |  |  |  |  |  |  |  |  |  |
| JP3 | *A*/*A*_r_ | 10/5.5 | 11/8.0 | 19/11.2 | 17/6.4 | 7/4.7 | 6/4.3 | 3/3.0 | 8/5.9 | 10.1/6.1 |
|  | *H*_O_ | 0.6042 | 0.3488 | 0.1395 | 0.5106 | 0.5909 | 0.1500 | 0.5000 | 0.5682 | 0.4265 |
|  | *H*_E_ | 0.7217 | 0.8460 | 0.9073 | 0.5797 | 0.6745 | 0.7038 | 0.6024 | 0.6844 | 0.7150 |
|  | *F*_IS_ | 0.16 | 0.60 | 0.85 | 0.12 | 0.13 | 0.70 | 0.17 | 0.17 | 0.36 |
|  | *P*_HW_ | **0.0539** | **0.0000** | **0.0000** | **0.0009** | 0.1730 | **0.0000** | 0.2648 | **0.0006** |  |
|  |  |  |  |  |  |  |  |  |  |  |
| KR | *A*/*A*_r_ | 18/11.3 | 17/11.1 | 8/5.8 | 15/10.6 | 30/15.8 | 11/8.4 | 8/5.9 | 9/7.1 | 14.5/9.5 |
|  | *H*_O_ | 0.3333 | 0.5172 | 0.1667 | 0.4286 | 0.5417 | 0.2500 | 0.3793 | 0.5714 | 0.3985 |
|  | *H*_E_ | 0.9134 | 0.9286 | 0.7828 | 0.8978 | 0.9690 | 0.8115 | 0.7562 | 0.8013 | 0.8576 |
|  | *F*_IS_ | 0.64 | 0.45 | 0.79 | 0.53 | 0.45 | 0.45 | 0.50 | 0.29 | 0.51 |
|  | *P*_HW_ | **0.0000** | **0.0000** | **0.0000** | **0.0000** | **0.0047** | **0.0000** | **0.0003** | **0.0004** |  |
|  |  |  |  |  |  |  |  |  |  |  |

Table S1 continued

| ID | Index |  |  |  | Microsatellite loci | | |  |  | Average |
| --- | --- | --- | --- | --- | --- | --- | --- | --- | --- | --- |
|  |  | Lf04 | Lf06 | Lf07 | Lf19 | Lf21 | Lf22 | Lf23 | Lf38 |  |
| CH1 | *A*/*A*_r_ | 31/12.6 | 12/8.4 | 17/8.2 | 22/8.1 | 33/13.1 | 12/7.4 | 8/6.1 | 9/6.7 | 18/8.8 |
|  | *H*_O_ | 0.4524 | 0.4634 | 0.4651 | 0.5556 | 0.5556 | 0.4250 | 0.5000 | 0.3824 | 0.4994 |
|  | *H*_E_ | 0.9337 | 0.8642 | 0.7335 | 0.7096 | 0.9348 | 0.7725 | 0.7038 | 0.7954 | 0.8059 |
|  | *F*_IS_ | 0.52 | 0.47 | 0.37 | 0.22 | 0.41 | 0.35 | 0.29 | 0.52 | 0.39 |
|  | *P*_HW_ | **0.0002** | **0.0000** | **0.0002** | **0.0000** | 0.0000 | **0.0000** | **0.0000** | **0.0000** |  |
|  |  |  |  |  |  |  |  |  |  |  |
| CH2 | *A*/*A*_r_ | 10/7.4 | 13/9.9 | 12/8.0 | 10/6.9 | 15/9.3 | 11/9.5 | 6/4.5 | 3/2.9 | 10/7.3 |
|  | *H*_O_ | 0.5500 | 0.8095 | 0.3810 | 0.5910 | 0.6364 | 0.5790 | 0.4546 | 0.2222 | 0.5280 |
|  | *H*_E_ | 0.7962 | 0.9083 | 0.6934 | 0.7040 | 0.8690 | 0.8848 | 0.4324 | 0.5222 | 0.7263 |
|  | *F*_IS_ | 0.32 | 0.11 | 0.46 | 0.16 | 0.27 | 0.15 | -0.05 | 0.58 | 0.25 |
|  | *P*_HW_ | 0.0240 | **0.0040** | **0.0014** | 0.5703 | 0.0000 | **0.0062** | 0.8107 | 0.0031 |  |
|  |  |  |  |  |  |  |  |  |  |  |
| CH3 | *A*/*A*_r_ | 23/11.1 | 19/10.5 | 10/5.3 | 14/7.0 | 12/6.5 | 13/8.3 | 8/4.3 | 6/4.1 | 13.1/7.1 |
|  | *H*_O_ | 0.4773 | 0.6136 | 0.5455 | 0.6977 | 0.5910 | 0.6744 | 0.4090 | 0.1316 | 0.5175 |
|  | *H*_E_ | 0.9122 | 0.9146 | 0.5802 | 0.6799 | 0.7908 | 0.7959 | 0.4276 | 0.5035 | 0.7006 |
|  | *F*_IS_ | 0.48 | 0.33 | 0.06 | -0.03 | 0.26 | 0.44 | 0.04 | 0.74 | 0.29 |
|  | *P*_HW_ | **0.0000** | **0.0000** | **0.5586** | 0.4736 | **0.0000** | **0.0000** | 0.0735 | **0.0000** |  |
|  |  |  |  |  |  |  |  |  |  |  |
| CH4 | *A*/*A*_r_ | 14/9.5 | 15/10.2 | 11/6.0 | 13/6.2 | 9/5.7 | 13/9.3 | 6/4.7 | 7/5.1 | 11/7.1 |
|  | *H*_O_ | 0.4643 | 0.4667 | 0.3103 | 0.5667 | 0.6552 | 0.5000 | 0.5000 | 0.1600 | 0.4529 |
|  | *H*_E_ | 0.8883 | 0.9147 | 0.4990 | 0.5085 | 0.7405 | 0.8825 | 0.5548 | 0.6237 | 0.7015 |
|  | *F*_IS_ | 0.48 | 0.49 | 0.38 | -0.11 | 0.12 | 0.50 | 0.10 | 0.75 | 0.34 |
|  | *P*_HW_ | **0.0000** | **0.0000** | **0.0050** | 0.9137 | **0.0026** | **0.0000** | 0.2592 | **0.0000** |  |
|  |  |  |  |  |  |  |  |  |  |  |
| CO | *A*/*A*_r_ | 8/7.4 | 10/7.9 | 4/3.8 | 5/4.2 | 6/5.8 | 6/5.0 | 9/8.1 | 6/5.4 | 6.8/6.0 |
|  | *H*_O_ | 0.0345 | 0.4828 | 0.1333 | 0.1667 | 0.0370 | 0.1667 | 0.2500 | 0.5000 | 0.2214 |
|  | *H*_E_ | 0.7992 | 0.6630 | 0.4186 | 0.2340 | 0.6660 | 0.3299 | 0.5987 | 0.5649 | 0.5343 |
|  | *F*_IS_ | 0.96 | 0.30 | 0.69 | 0.30 | 0.95 | 0.50 | 0.59 | 0.12 | 0.59 |
|  | *P*_HW_ | **0.0000** | **0.0000** | **0.0000** | 0.2719 | **0.0000** | 0.0031 | **0.0000** | 0.0513 |  |
|  |  |  |  |  |  |  |  |  |  |  |
| RB | *A*/*A*_r_ | 7/6.2 | 12/9.5 | 3/3.0 | 6/4.9 | 9/7.6 | 6/4.9 | 7/6.1 | 5/5.0 | 6.9/5.9 |
|  | *H*_O_ | 0.1290 | 0.3750 | 0.2000 | 0.1111 | 0.0645 | 0.1613 | 0.1667 | 0.6207 | 0.2285 |
|  | *H*_E_ | 0.5643 | 0.6940 | 0.4333 | 0.2739 | 0.6536 | 0.3681 | 0.5588 | 0.7641 | 0.5387 |
|  | *F*_IS_ | 0.77 | 0.51 | 0.54 | 0.60 | 0.90 | 0.57 | 0.71 | 0.19 | 0.59 |
|  | *P*_HW_ | **0.0000** | **0.0000** | **0.0002** | **0.0002** | **0.0000** | **0.0005** | **0.0000** | 0.0023 |  |
|  |  |  |  |  |  |  |  |  |  |  |
| IT | *A*/*A*_r_ | 13/11.0 | 8/6.8 | 3/3.0 | 6/5.3 | 7/6.8 | 8/7.1 | 9/7.1 | 8/7.1 | 7.8/6.8 |
|  | *H*_O_ | 0.3000 | 0.4815 | 0.0667 | 0.1739 | 0.1667 | 0.3214 | 0.2143 | 0.5172 | 0.2802 |
|  | *H*_E_ | 0.8435 | 0.7254 | 0.4249 | 0.2821 | 0.8011 | 0.5539 | 0.3831 | 0.7187 | 0.5916 |
|  | *F*_IS_ | 0.65 | 0.36 | 0.85 | 0.39 | 0.80 | 0.47 | 0.51 | 0.28 | 0.54 |
|  | *P*_HW_ | **0.0000** | **0.0003** | **0.0000** | 0.0056 | **0.0000** | **0.0000** | **0.0000** | 0.0216 |  |
|  |  |  |  |  |  |  |  |  |  |  |

| ID | Index |  |  |  | Microsatellite loci | | |  |  | Average |
| --- | --- | --- | --- | --- | --- | --- | --- | --- | --- | --- |
|  |  | Lf04 | Lf06 | Lf07 | Lf19 | Lf21 | Lf22 | Lf23 | Lf38 |  |
| YR | *A*/*A*_r_ | 13/11.8 | 9/7.9 | 3/2.7 | 4/3.8 | 8/7.7 | 5/4.6 | 6/5.8 | 6/4.4 | 6.8/6.1 |
|  | *H*_O_ | 0.0800 | 0.4348 | 0.0800 | 0.1111 | 0.1429 | 0.0417 | 0.0741 | 0.1923 | 0.1446 |
|  | *H*_E_ | 0.8833 | 0.6609 | 0.2800 | 0.2411 | 0.8240 | 0.3661 | 0.6178 | 0.4223 | 0.5369 |
|  | *F*_IS_ | 0.91 | 0.43 | 0.72 | 0.54 | 0.83 | 0.89 | 0.88 | 0.67 | 0.73 |
|  | *P*_HW_ | **0.0000** | 0.0030 | **0.0010** | 0.0014 | **0.0000** | **0.0000** | **0.0000** | **0.0000** |  |
|  |  |  |  |  |  |  |  |  |  |  |
| YD | *A*/*A*_r_ | 5/5.0 | 5/4.6 | 2/2.0 | 10/8.7 | 10/9.1 | 7/6.4 | 6/5.6 | 5/4.6 | 6.3/5.8 |
|  | *H*_O_ | 0.1667 | 0.1364 | 0.0400 | 0.2174 | 0.0345 | 0.0400 | 0.0455 | 0.3462 | 0.1283 |
|  | *H*_E_ | 0.7207 | 0.5708 | 0.1151 | 0.4860 | 0.6818 | 0.6114 | 0.5085 | 0.6154 | 0.5387 |
|  | *F*_IS_ | 0.77 | 0.77 | 0.66 | 0.56 | 0.96 | 0.94 | 0.91 | 0.44 | 0.75 |
|  | *P*_HW_ | **0.0000** | **0.0000** | 0.0630 | **0.0000** | **0.0000** | **0.0000** | **0.0000** | 0.0028 |  |
|  |  |  |  |  |  |  |  |  |  |  |
| SA | *A*/*A*_r_ | 10/8.9 | 8/6.6 | 3/3.0 | 5/3.7 | 9/7.9 | 7/5.7 | 7/5.4 | 8/7.6 | 7.1/6.1 |
|  | *H*_O_ | 0.4333 | 0.3125 | 0.0294 | 0.1482 | 0.0909 | 0.2424 | 0.1250 | 0.4546 | 0.2295 |
|  | *H*_E_ | 0.7927 | 0.6364 | 0.2884 | 0.1440 | 0.7324 | 0.4592 | 0.3373 | 0.7664 | 0.5196 |
|  | *F*_IS_ | 0.46 | 0.5 | 0.95 | -0.03 | 0.88 | 0.48 | 0.63 | 0.41 | 0.54 |
|  | *P*_HW_ | **0.0000** | **0.0000** | **0.0000** | 1.0000 | **0.0000** | **0.0000** | **0.0000** | **0.0000** |  |
|  |  |  |  |  |  |  |  |  |  |  |
| SO | *A*/*A*_r_ | 6/5.6 | 11/9.3 | 7/5.3 | 4/3.8 | 7/5.7 | 4/3.4 | 6/5.4 | 9/8.4 | 6.8/5.9 |
|  | *H*_O_ | 0.2000 | 0.4231 | 0.1471 | 0.0345 | 0.1290 | 0.1333 | 0.1852 | 0.5000 | 0.2190 |
|  | *H*_E_ | 0.6198 | 0.7376 | 0.4056 | 0.3612 | 0.7689 | 0.2955 | 0.4661 | 0.8220 | 0.5596 |
|  | *F*_IS_ | 0.68 | 0.45 | 0.71 | 0.91 | 0.83 | 0.56 | 0.61 | 0.40 | 0.64 |
|  | *P*_HW_ | **0.0000** | **0.0000** | **0.0000** | **0.0000** | **0.0000** | 0.0034 | **0.0002** | **0.0000** |  |
|  |  |  |  |  |  |  |  |  |  |  |
| UR | *A*/*A*_r_ | 6/5.7 | 7/6.8 | 4/3.7 | 4/4.0 | 5/5.0 | 3/2.8 | 5/4.7 | 8/7.7 | 5.3/5.1 |
|  | *H*_O_ | 0.0800 | 0.3462 | 0.1600 | 0.0455 | 0.1818 | 0.0833 | 0.2400 | 0.5000 | 0.2046 |
|  | *H*_E_ | 0.5706 | 0.7330 | 0.3200 | 0.4789 | 0.7452 | 0.2598 | 0.5559 | 0.7231 | 0.5483 |
|  | *F*_IS_ | 0.86 | 0.53 | 0.50 | 0.90 | 0.76 | 0.69 | 0.57 | 0.31 | 0.64 |
|  | *P*_HW_ | **0.0000** | **0.0000** | **0.0004** | **0.0000** | **0.0000** | **0.0004** | **0.0000** | 0.0316 |  |
|  |  |  |  |  |  |  |  |  |  |  |
| RT | *A*/*A*_r_ | 7/6.2 | 10/8.2 | 7/5.6 | 6/4.9 | 6/5.7 | 7/6.0 | 9/7.3 | 8/7.3 | 7.5/6.4 |
|  | *H*_O_ | 0.1333 | 0.3333 | 0.0667 | 0.1600 | 0.0000 | 0.1429 | 0.2333 | 0.3667 | 0.1795 |
|  | *H*_E_ | 0.4542 | 0.7000 | 0.3571 | 0.2604 | 0.7100 | 0.4792 | 0.5627 | 0.7068 | 0.5288 |
|  | *F*_IS_ | 0.71 | 0.57 | 0.82 | 0.39 | 1.00 | 0.76 | 0.65 | 0.49 | 0.68 |
|  | *P*_HW_ | **0.0000** | **0.0000** | **0.0000** | 0.0249 | **0.0000** | **0.0000** | **0.0000** | **0.0000** |  |
|  |  |  |  |  |  |  |  |  |  |  |
| EC | *A*/*A*_r_ | 11/9.7 | 12/10.1 | 3/2.7 | 7/5.1 | 7/6.6 | 6/5.6 | 7/5.8 | 6/5.0 | 7.4/6.2 |
|  | *H*_O_ | 0.0882 | 0.4412 | 0.0000 | 0.2105 | 0.0645 | 0.1035 | 0.0606 | 0.4000 | 0.1711 |
|  | *H*_E_ | 0.8635 | 0.7050 | 0.4132 | 0.2895 | 0.7081 | 0.5475 | 0.4960 | 0.5836 | 0.5758 |
|  | *F*_IS_ | 0.90 | 0.32 | 1.00 | 0.28 | 0.91 | 0.82 | 0.88 | 0.32 | 0.68 |
|  | *P*_HW_ | **0.0000** | 0.0073 | **0.0000** | 0.0374 | **0.0000** | **0.0000** | **0.0000** | 0.0413 |  |
|  |  |  |  |  |  |  |  |  |  |  |

Table S1 continued

| ID | Index |  |  |  | Microsatellite loci | | |  |  | Average |
| --- | --- | --- | --- | --- | --- | --- | --- | --- | --- | --- |
|  |  | Lf04 | Lf06 | Lf07 | Lf19 | Lf21 | Lf22 | Lf23 | Lf38 |  |
| TI | *A*/*A*_r_ | 8/6.7 | 11/8.6 | 5/3.9 | 7/4.9 | 11/10.0 | 6/5.4 | 9/6.8 | 7/6.3 | 8.0/6.6 |
|  | *H*_O_ | 0.2051 | 0.4054 | 0.0526 | 0.0882 | 0.1212 | 0.1563 | 0.0857 | 0.4000 | 0.1893 |
|  | *H*_E_ | 0.6956 | 0.6027 | 0.2432 | 0.2476 | 0.8611 | 0.4911 | 0.3818 | 0.7023 | 0.5282 |
|  | *F*_IS_ | 0.70 | 0.40 | 0.89 | 0.65 | 0.86 | 0.79 | 0.87 | 0.43 | 0.69 |
|  | *P*_HW_ | **0.0000** | **0.0000** | **0.0000** | **0.0000** | **0.0000** | **0.0000** | **0.0000** | **0.0000** |  |
|  |  |  |  |  |  |  |  |  |  |  |
| QU | *A*/*A*_r_ | 10/8.2 | 8/7.3 | 7/5.8 | 5/4.2 | 9/8.0 | 8/6.8 | 8/6.9 | 6/5.7 | 7.6/6.6 |
|  | *H*_O_ | 0.2000 | 0.2778 | 0.0263 | 0.0556 | 0.2874 | 0.2222 | 0.2308 | 0.3214 | 0.2027 |
|  | *H*_E_ | 0.8207 | 0.6377 | 0.3926 | 0.2567 | 0.6901 | 0.5430 | 0.5881 | 0.5240 | 0.5566 |
|  | *F*_IS_ | 0.76 | 0.52 | 0.96 | 0.79 | 1.00 | 0.59 | 0.61 | 0.39 | 0.70 |
|  | *P*_HW_ | **0.0000** | **0.0000** | **0.0000** | **0.0000** | **0.0000** | **0.0000** | **0.0000** | 0.0011 |  |
|  |  |  |  |  |  |  |  |  |  |  |
| SL | *A*/*A*_r_ | 9/8.2 | 11/8.8 | 8/6.0 | 4/3.7 | 6/6.0 | 5/4.8 | 5/4.5 | 7/7.0 | 6.9/6.1 |
|  | *H*_O_ | 0.3333 | 0.3103 | 0.1852 | 0.1905 | 0.0000 | 0.1429 | 0.1071 | 0.5263 | 0.2245 |
|  | *H*_E_ | 0.7072 | 0.6370 | 0.4570 | 0.2973 | 0.7758 | 0.4526 | 0.3214 | 0.8037 | 0.5565 |
|  | *F*_IS_ | 0.53 | 0.45 | 0.70 | 0.37 | 1.00 | 0.69 | 0.67 | 0.35 | 0.60 |
|  | *P*_HW_ | **0.0000** | **0.0000** | **0.0000** | 0.0074 | **0.0000** | **0.0000** | **0.0000** | **0.0008** |  |
|  |  |  |  |  |  |  |  |  |  |  |
| MA | *A*/*A*_r_ | 6/5.3 | 9/8.5 | 6/4.7 | 5/4.5 | 7/5.7 | 6/4.9 | 8/6.0 | 5/4.6 | 6.5/5.5 |
|  | *H*_O_ | 0.0606 | 0.5484 | 0.1613 | 0.1290 | 0.1290 | 0.2121 | 0.1818 | 0.3077 | 0.2162 |
|  | *H*_E_ | 0.6783 | 0.8271 | 0.4537 | 0.3681 | 0.7742 | 0.4317 | 0.3534 | 0.4253 | 0.5390 |
|  | *F*_IS_ | 0.85 | 0.22 | 0.67 | 0.78 | 0.80 | 0.65 | 0.51 | 0.47 | 0.62 |
|  | *P*_HW_ | **0.0000** | **0.0001** | **0.0000** | **0.0000** | **0.0000** | **0.0000** | **0.0000** | 0.0768 |  |
|  |  |  |  |  |  |  |  |  |  |  |

Table S1 continued
