# Supplementary material for: Genetic Diversity in Introduced Golden Mussel Populations Corresponds to Vector Activity
Source: PLoS One. 2013 Mar 22;8(3):e59328. doi: 10.1371/journal.pone.0059328 (PMC3606440; doi:10.1371/journal.pone.0059328)
Supplement: Table S2 — Comparison of microsatellite-based genetic features of the three highly invasive freshwater mussels, zebra mussel Dreissena polymorpha, quagga mussel Dreissena rostriformis bugensis, and golden mussel Limnoperna fortunei. (DOC) [file pone.0059328.s003.doc]

**Table S2.** Comparison of microsatellite-based genetic features of the three highly invasive freshwater mussels, zebra mussel *Dreissena polymorpha*, quagga mussel *Dreissena rostriformis bugensis*, and golden mussel *Limnoperna fortunei.*

| Feature | Zebra mussel  *Dreissena polymorpha* | Quagga mussel  *Dreissena rostriformis bugensis* | Golden mussel  *Limnoperna fortunei* |
| --- | --- | --- | --- |
| Native range  (Reference) | Ponto-Caspian Region  (Son, 2007) | Dnieper Delta  (Son, 2007) | China and southeast Asia  (Ricciardi, 1998) |
| Native pop. surveyed  (genetic diversity) | Romania  (*H*_E_ = 0.894) | Black and Caspian Seas region  (*H*_E_ = 0.778 – 0.893) | China and Korea  (*H*_E_ = 0.701 – 0.858) |
| Introduced pop. surveyed  (genetic diversity) | Great Lakes  (*H*_E_ = 0.878 – 0.888)  Europe  (*H*_E_ = 0.790 – 0.940) | Great Lakes  (*H*_E_ = 0.802 – 0.931)  Black and Caspian Seas region  (*H*_E_ = 0.796 – 0.931)  Germany  (*H*_E_ = 0.873 – 0.931) | South America  (*H*_E_ = 0.485 – 0.621)  Japan  (*H*_E_ = 0.715 – 0.746)  Taiwan  (*H*_E_ = 0.667 – 0.743) |
| Genetic differentiation  within native pop. | *F*_ST_ = 0.051 | *F*_ST_ = 0.008 – 0.030 | *F*_ST_ = 0.051 (= 0.017 – 0.096) |
| Genetic differentiation  within introduced pop. | *F*_ST_ = -0.007 – 0.032  *F*_ST_ = 0.006 – 0.263 | *F*_ST_ = 0 – 0.026  *F*_ST_ = 0.008–0.267 | *F*_ST_ = 0.004 – 0.107 |
| Genetic differentiation between  introduced and native pop. | *F*_ST_ = 0.008 – 0.054 | *F*_ST_ = 0.002 – 0.035 | *F*_ST_ = 0.037 – 0.299 |
| Dispersal dynamics suggested by  genetic analyses in invaded range | Ship-mediated dispersal &  downstream movement of larvae | Ship-mediated dispersal | Ship-mediated dispersal  Ship-mediated “jump” dispersal |
| References | Müller *et al*., 2002;  Astanei *et al*., 2005  Brown & Sptepien 2010 | Wilson *et al*., 1999;  Therriault *et al*., 2005  Imo *et al*., 2010  Brown & Sptepien 2010 | Zhan *et al*. 2012  This study |
